# Resolutions of Respect to Dr. Eames

**Published:** 1894-05

**Authors:** 


### RESOLUTIONS OF RESPECT TO DR. EAMES.

At the regular meeting of the Chicago Dental Society, April 3, 1894, the following resolutions were passed:

Whereas, It has come to the knowledge of the members of the Chicago Dental Society that the dental profession is called to mourn the loss of one of its most distinguished and honored members. Therefore, be it

*Resolved*, That in the decease of Professor W. H. Eames the world has lost one of its foremost educators, his family a devoted husband and father, and the profession a sincere, earnest, and useful devotee. It is fitting that the Society should pay its tribute of respect to one who has so long and honorably filled the position, before the world, and we mourn his death as a personal loss to each and all of us. Be it further

*Resolved*, That a copy of these lines be transmitted to the family of the deceased, and a further copy be sent to the journals for publication.

A. W. Harlan.
Truman W. Brophy.
Edgar D. Swain.

